# Chorea-Acanthocytosis Presenting as Autosomal Recessive Epilepsy in a Family With a Novel *VPS13A* Mutation

**DOI:** 10.3389/fneur.2018.01168

**Published:** 2019-01-09

**Authors:** Juliane Weber, Lars Frings, Michel Rijntjes, Horst Urbach, Judith Fischer, Cornelius Weiller, Philipp T. Meyer, Stephan Klebe

**Affiliations:** ^1^Department of Neurology, Faculty of Medicine, Medical Center, University of Freiburg, Freiburg, Germany; ^2^Department of Nuclear Medicine, Faculty of Medicine, Medical Center, University of Freiburg, Freiburg, Germany; ^3^Department of Neuroradiology, Faculty of Medicine, Medical Center, University of Freiburg, Freiburg, Germany; ^4^Department of Human Genetics, Faculty of Medicine, Medical Center, University of Freiburg, Freiburg, Germany; ^5^Department of Neurology, University Hospital of Essen, Essen, Germany

**Keywords:** chorea-acanthocytosis, neuro-acanthocytosis, *VPS13A*, genome sequencing, autosomal recessive epilepsy, FDG-PET

## Abstract

Chorea-acanthocytosis (ChAc) is a rare, adult-onset disease usually characterized by, hence the name, a movement disorder and acanthocytosis in the blood. It is caused by mutations of the *VPS13A* gene with an autosomal recessive transmission. We report a consanguineous Turkish family with a different and informative clinical and diagnostic course. Three siblings developed seizures and the index patient had been diagnosed with bilateral temporal lobe epilepsy. A key finding, however, was the basal ganglia involvement in neuroimaging although no movement disorder was present. [^18^F]FDG-PET showed a prominent decline in striatal glucose metabolism at 31 years of age and [^123^I]FP-CIT-SPECT revealed a moderate loss of striatal dopamine transporter availability. The family was referred for genetic testing and exome sequencing detected a homozygous novel truncating mutation c.4326 T>A (p.Tyr1442^*^) in *VPS13A* in all affected siblings. With this case, we present autosomal recessive epilepsy as the predominant phenotype of ChAc with a new homozygous *VPS13A* mutation and provide pathological structural and molecular neuroimaging findings.

## Introduction

Neuroacanthocytosis is a superordinate term for a group of rare syndromes that are characterized by neurological symptoms in combination with spiky deformed red blood cells (acanthocytes). This report focuses on Chorea-acanthocytosis (ChAc) [Online Mendelian Inheritance in Man (OMIM) #200150], which is an orphan disease with estimated 1,000 affected individuals worldwide caused by autosomal-recessive mutations in the *VPS13A* (vacuolar protein sorting 13 homolog A) gene on chromosome 9q (1). The disease runs a progressive course, causes of increased mortality may be declined motor functions correlating with risk conditions such as dysphagia, but also sudden unexpected deaths have been described (2, 3). So far, there is no causative treatment.

The suspicious coexistence of acanthocytosis and movement disorders was first reported in the 1970s by Levine and Critchley (4, 5) and has since been accepted as the prominent feature of the disease. However, with our case report we describe a rare clinical phenotype of ChAc lacking obvious movement disorders, suggesting a broader variety of clinical presentations. Diagnostic tools have to meet these challenges to establish the correct diagnosis, we here suggest molecular neuroimaging as a key method.

## Case Report

The male index patient first presented at 25 years of age with two unprovoked bilateral tonic clonic seizures. There was no medical history. The brain MRI scan and EEG at that age were normal and no treatment was initiated due to the patient's reluctance and infrequent events. However, the patient had ongoing seizures now presenting as partial epilepsy. He described reoccurring feelings of sudden dizziness that were diagnosed as a vertiginous aura. Furthermore, he had dyscognitive seizures in which he showed either (a) unresponsiveness and reciting of Turkish prayers, (b) oral automatisms, or (c) fiddling with the hands and uttering sounds—all partly evolving into tonic clonic seizures. Video-EEG now showed local spike-wave complexes both in the right and left temporal lobe. In accordance with the semiology of seizure, the diagnosis of bilateral temporal lobe epilepsy was made and medication with levetiracetam was started.

**Figure 1:**
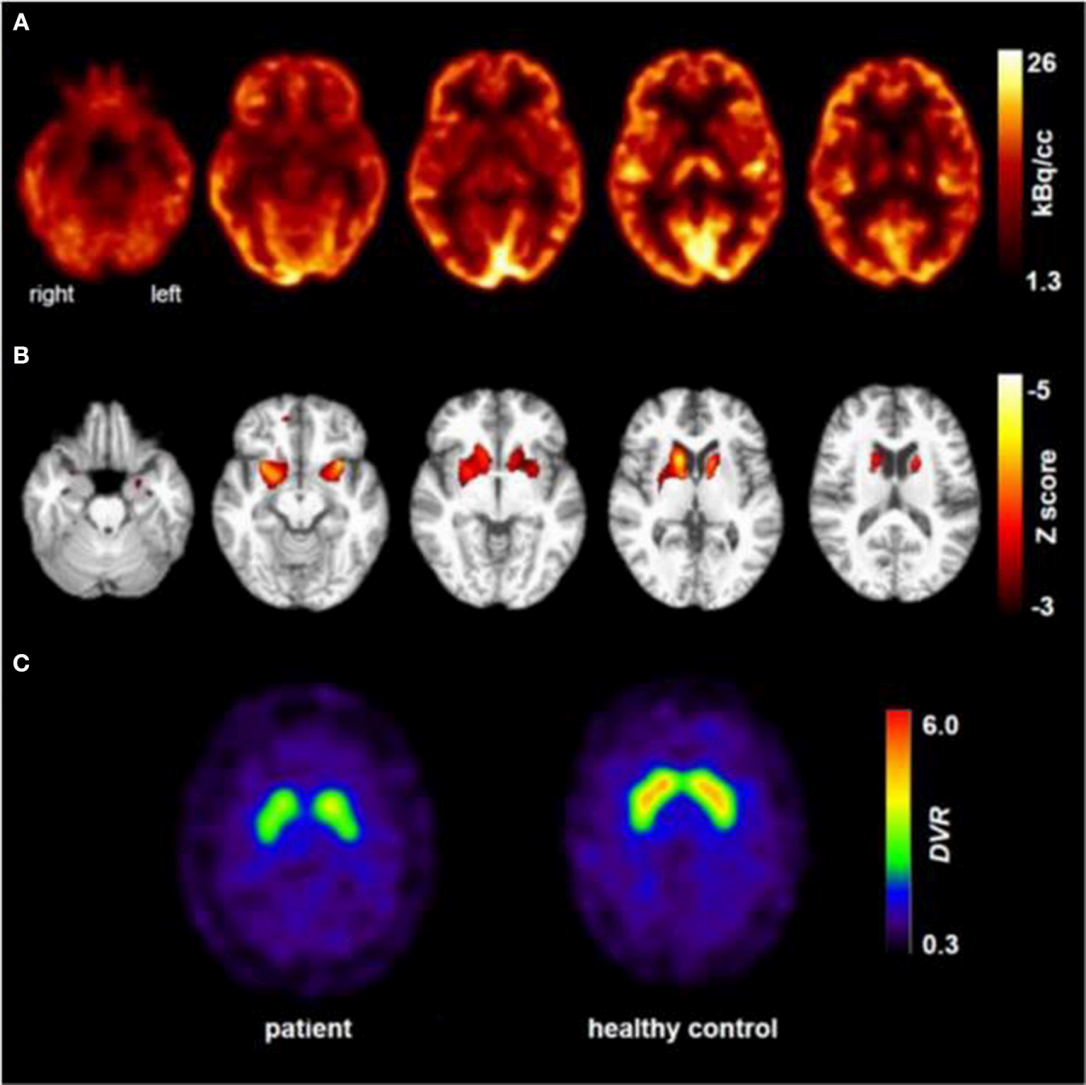
Results of molecular imaging studies. Transaxial [^18^F]FDG-PET images **(A)** showed not only a mild bilateral mesiotemporal hypometabolism (in line with mesiotemporal seizure origin) but also a marked striatal hypometabolism that was found to be highly significant compared to healthy controls [**(B)**; results of a statistical parametric mapping analysis comparing the patient's scan to those of 10 age-matched healthy controls; clusters of voxels with significant hypometabolism (voxel threshold: *p* < 0.001, cluster extent threshold *k* > 20 voxels) are color-coded as Z score and overlaid onto the spatially-normalized MRI scan of the patient]. An additional [^123^I]FP-CIT-SPECT examination **(C)** also revealed a moderate loss of striatal dopamine transporters, witnessing a decline of nigrostriatal integrity (an age-matched healthy control is shown for comparison; *DVR*, distribution volume ratio).

**Figure 2:**
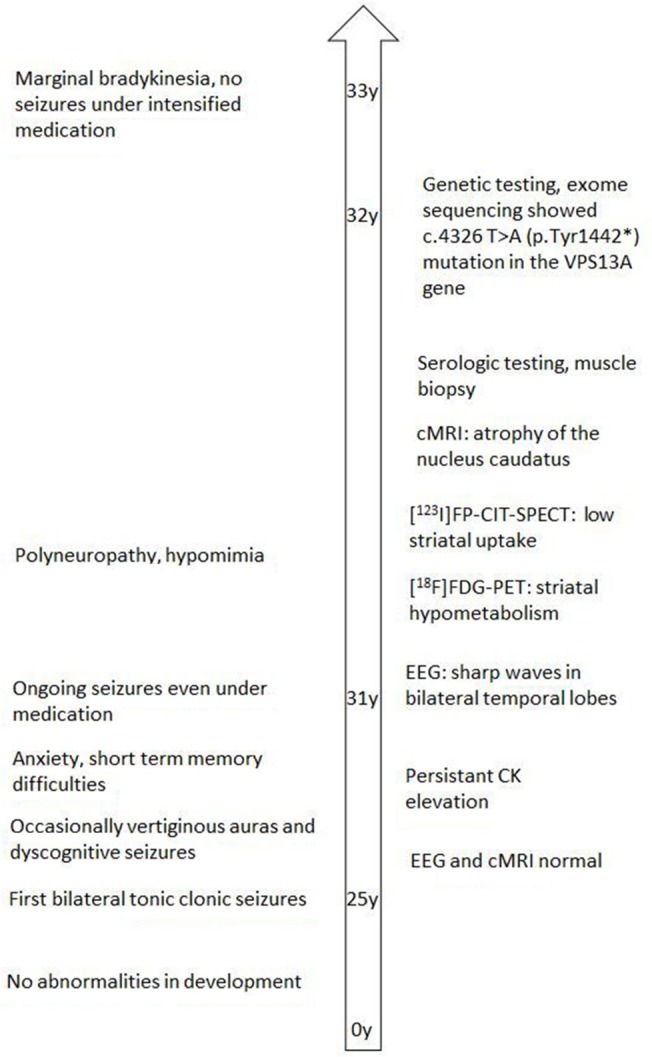
Clinical and diagnostic course of the index patient.

**Figure 3:**
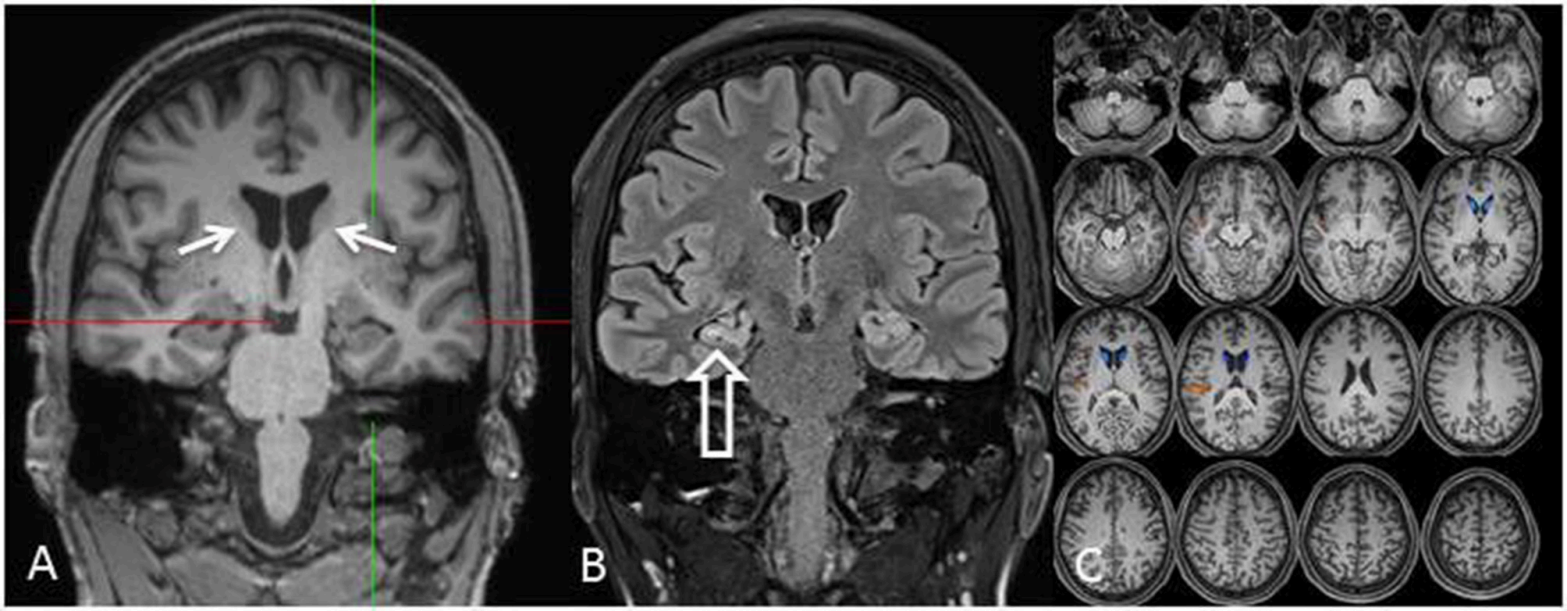
MRI [coronal reformation of a MPRAGE sequence, **(A)**; coronal FLAIR sequence, **(B)**; z-score map of a combined voxel and region based (CVR) analysis, **(C)**] shows discrete bilateral caudate head atrophy [**(A)**: arrows] and a smaller and hyperintense right-sided hippocampus indicating right-sided hippocampal sclerosis [**(B)**: hollow arrow]. The left hippocampus is somewhat hyperintense but not atrophic. Bilateral caudate head atrophy is confirmed with CVR analysis, in which blue colors show decreased gray and red colors increased cerebrospinal fluid volume, respectively **(C)**.

At the age of 31 seizures were not entirely suppressed, as part of further diagnostics of temporal lobe epilepsy, the patient underwent a [^18^F]FDG-PET (Figure 1) which showed a bilateral mesiotemporal hypometabolism, in line with the mesiotemporal seizure origin. However, there was also a marked, highly unusual striatal hypometabolism which raised the suspicion of a neurodegenerative movement disorder. Consequently, an extensive follow-up evaluation was initiated (see Figure 2). A [^123^I]FP-CIT-SPECT was performed which yielded a moderately bilateral loss of striatal dopamine transporter availability, in line with a decline of nigrostriatal integrity (Figure 1). A high-resolution MRI now presented bilateral atrophy of the nucleus caudatus (Figure 3). Neurological examination showed a discrete bound gait pattern and hypomimia but no other affection of the extrapyramidal motor system and no bulbar symptoms like feeding dystonia. Apart from low reflex status on the lower limbs, for which nerve conduction studies confirmed a sensorimotor axonal polyneuropathy, the neurological examination was normal. Neuropsychological symptoms included aspects of an anxiety disorder and the patient reported difficulties with short term memory loss. Neuropsychological testing however did not confirm manifest mnestic malfunction but rather an unspecific distraction, which might additionally have been enhanced by insufficient seizure suppression at the time. Laboratory testing was negative for any symptomatic epilepsy (i.e., autoimmune-encephalitis antibodies, antineuronal antibodies). Cerebrospinal fluid showed no signs of inflammation but a moderate protein increase (765 mg/l). Analysis of biogenic amines in the cerebrospinal fluid revealed an elevation of glutamine which we associated with recent seizures. In the peripheral blood, 0.4% of acanthocytes were found. Serologic testing for Wilson's disease was negative. Noticeable was a persistent elevation of creatine kinase (range: 1,000–3,000 U/l) which led to the suspected diagnosis of a mitochondrial disorder. For further investigations, an ischemic lactate test was performed which showed normal results. A muscle biopsy of the M. gastrocnemicus was performed, but analysis of the mitochondrial function and respiratory chain was normal.

**Figure 4:**
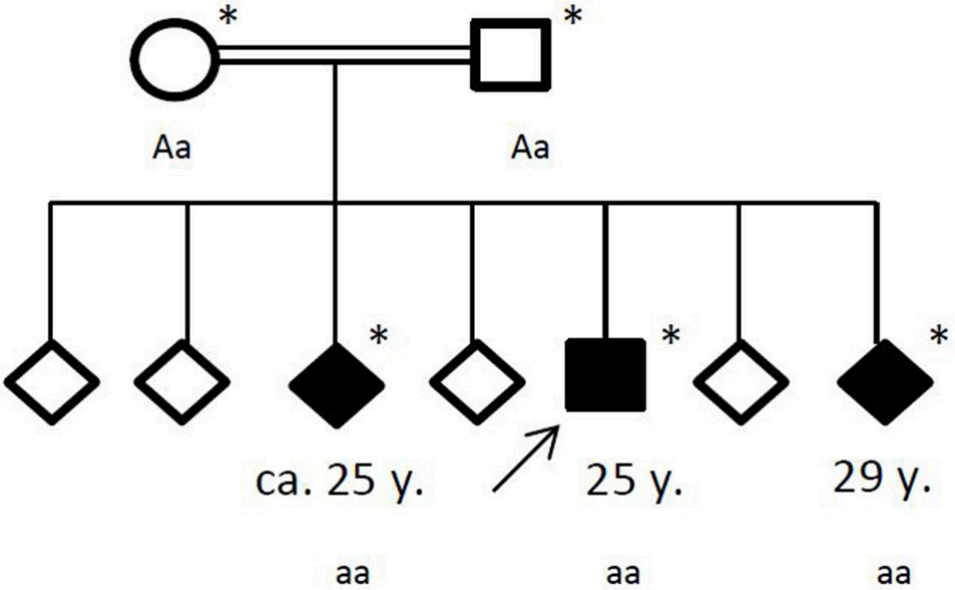
Pedigree of the affected family, with age of first epileptic seizure in years (y). Arrow: index patent. Asterisk: genetic testing performed. Aa, heterozygous; aa, homozygous for c.4326 T>A (p.Tyr1442*).

Family history revealed a consanguinity of his parents (first cousins), who themselves had no significant medical history. Of his six siblings, two (between the ages of 20–30 years) had by now also suffered from general seizures (see Figure 4). Medical records of his siblings were not available to us.

Under the hypothesis of a hereditary neurodegenerative movement disorder, the patient and his family were referred for genetic testing. Exome sequencing revealed a homozygous novel truncating mutation c.4326 T>A (p.Tyr1442^*^) in the ChAc gene *VPS13A* in the three affected siblings. The consanguine parents were both detected heterozygous. No other variants or mutations were identified in the exome sequencing.

In fact, in a follow-up at the age of 33 years, the index patient still only had marginal extrapyramidal symptoms and none were present in any of his sisters. He now gave the impression of slight rigidity only under priming on the right upper limb, and a mild bradykinesia of the upper limbs. Under therapy with levetiracetam and lacosamide, the patient has been seizure free.

## Discussion

With the present case, we demonstrate that ChAc can clinically manifest with temporal lobe epilepsy lacking any significant motor symptoms due to a new corresponding mutation in the *VPS13A* gene.

The disease course is typically characterized by a progressive movement disorder (including chorea, dystonia, parkinsonism), cognitive and psychiatric changes and myopathic symptoms with serological markers of acanthocytosis and hyperCKemia. Seizures have previously been noted as a symptom of ChAc (6) but movement disorders are still considered the key symptom. Evidence arises that epilepsy, and more specifically epilepsy originating in the temporal lobe, might be an underestimated phenotype of ChAc. Peluso et al. reported a patient similar to ours, who, even at the age of 46 years, did not show movement disorders while presenting neuroimaging findings indicative of basal ganglia involvement (7). In accordance with that, Scheid et al. described three patients who suffered from mesial temporal lobe epilepsy and sclerosis (based on MRI studies) as the predominant symptom and were diagnosed with ChAc (8). Furthermore, Mente et al. recently provided autopsy results of a ChAc patient who displayed not only basal ganglia atrophy but also hippocampal sclerosis (9). In line with our case, bilateral involvement of the hippocampus seems to be a common finding (10, 11). The exact relation between seizures and sclerosis is, however, still a question of hen or egg. The correlating role of chorein in the structural development of the hippocampus is not yet fully understood. Interestingly, in a mouse model of ChAc structural change was seen, as hippocampal protein expression of the GABA (A) receptor gamma 2 and its anchoring protein chorein were increased (12).

In clinical routine, this phenotype can still be challenging in finding the correct diagnosis. The first crucial hint toward a neurodegenerative disease in our patient was a prominent decline in striatal glucose metabolism and a moderate decline in nigrostriatal integrity on [^18^F]FDG-PET and [^123^I]FP-CIT-SPECT, respectively. The small collection of functional imaging results, based on case series description, show that alterations in metabolism and dopaminergic dysfunction mostly occur in the caudate nucleus and putamen (13). Brain MRI revealed atrophy of the nucleus caudatus during the course of the disease, which has been described as a typical finding in ChAc (14). Accordingly, distinct neurologic features usually include chorea, feeding dystonia, orofaciolingual dyskinesias, and tics, alongside other symptoms of basal ganglia affection such as parkinsonism and dystonia (3). It is tempting to speculate that the observed striatal changes in our patient were still below the threshold of causing symptoms (i.e., prodromal imaging findings), which may ultimately occur as the disease progresses. We therefore encourage structural and molecular imaging as a sensitive diagnostic tool in this orphan disease.

In ChAc, there are descriptions of acanthocyte counts between 5 and 50% (3), but there are a few case reports that demonstrate that acanthocytes may appear only late in the course of the disease (15), might be very low, or even be fully absent (16). Therefore, acanthocytes should not be considered mandatory for making the diagnosis. Additionally epilepsy might delay diagnosis as it disguises other typical symptoms of ChAc. In particular, psychiatric symptoms such as personality changes and cognitive deterioration, which are very common, can be misinterpreted and attributed to drug-related side effects (i.e., of levetiracetam) or seizure-related. Secondary hyperCKemia is seen after general tonic clonic seizures and a persistent elevation might be overlooked.

Our case also highlights the crucial benefit of exome sequencing to establish a correct diagnosis, especially in rare diseases or when symptoms are vague. The family of mammalian *VPS13* (A–D) genes is of rising interest and mutations have been identified in other neurological diseases such as autosomal recessive Parkinson's disease or autosomal recessive spinocerebellar ataxia (17). Recessive mutations in *VPS13D* cause childhood onset movement disorders (18). The underlying pathophysiology of ChAc is not yet fully deciphered, but it was shown that *VPS13A* encodes the protein chorein which is expressed ubiquitously in the brain and in a vast number of other tissues (19). Studies reveal that it participates in signaling pathways that regulate cytoskeletal architecture, exocytosis, and cell survival (20). Another mutation that has been described to present with a seizure dominated phenotype in 9 patients with ChAc is the c.2343del mutation in the *VPS13A* gene (21). It is reasonable to assume that specific mutations in the same gene result in a certain aberration of chorein that causes a unique phenotype, for example by affecting different areas of the brain. However, the underlying pathophysiology is still only speculative and further documentation of the causative mutations will be of interest. We show here that the truncating mutation c.4326 T>A (p.Tyr1442^*^), which has not been described before, results in a similar phenotype with predominant epilepsy in all affected family members.

## Concluding Remarks

Epilepsy (particularly bilateral temporal lobe epilepsy) is likely to be a predominant phenotype of ChAc. Therefore, a search for additional red flags (such as hyperCKemia, family history, polyneuropathy) should be carefully performed. In suspicious cases search for acanthocytes in the blood and brain MRI should be added as these tools are widely available. If in doubt diagnostics should be complemented with [^18^F]FDG-PET and/or [^123^I]FP-CIT-SPECT as they are sensitive in detecting striatal involvement. In autosomal recessive epilepsies with indicative findings for a neurodegenerative disease *VPS13A* single gene test or chorein Western blot should then be used to establish the right diagnosis. The latter should also be considered in other neurodegenerative disorders without genetically defined etiology.

## Ethics Statement

Written informed consent was obtained from the patient for participation in the study. Written informed consent was obtained from the patient for the publication of this case report.

## Author Contributions

JW, MR, and SK took part in patient management. JW wrote the first draft of the manuscript. LF, HU, and PM prepared figures. All authors contributed to manuscript revision, read and approved the submitted version.

### Conflict of Interest Statement

HU is shareholder of the Veobrain Gmbh, a spin-off of the University Medical Center Freiburg. The remaining authors declare that the research was conducted in the absence of any commercial or financial relationships that could be construed as a potential conflict of interest.
